# Information in morphological characters

**DOI:** 10.1002/ece3.7874

**Published:** 2021-08-04

**Authors:** Congyu Yu, Qigao Jiangzuo, Emanuel Tschopp, Haibing Wang, Mark Norell

**Affiliations:** ^1^ Division of Paleontology American Museum of Natural History New York NY USA; ^2^ Department of Earth and Environmental Sciences Columbia University New York NY USA; ^3^ Key Laboratory of Vertebrate Evolution and Human Origins of Chinese Academy of Sciences Institute of Vertebrate Paleontology and Paleoanthropology Chinese Academy of Sciences Beijing China; ^4^ Key Laboratory of Orogenic Belts and Crustal Evolution School of Earth and Space Sciences Peking University Beijing China; ^5^ CAS Center for Excellence in Life and Paleoenvironment Beijing China; ^6^ Center of Natural History University of Hamburg Hamburg Germany

**Keywords:** character weighting, information theory, morphology, systematics

## Abstract

The construction of morphological character matrices is central to paleontological systematic study, which extracts paleontological information from fossils. Although the word information has been repeatedly mentioned in a wide array of paleontological systematic studies, its meaning has rarely been clarified nor specifically defined. It is important, however, to establish a standard to measure paleontological information because fossils are hardly complete, rendering the recognition of homologous and homoplastic structures difficult. Here, based on information theory, we show the deep connections between paleontological systematic study and communication system engineering. Information is defined as the decrease of uncertainty and it is the information in morphological characters that allows distinguishing operational taxonomic units (OTUs) and reconstructing evolutionary history. We propose that concepts in communication system engineering such as source coding and channel coding, correspond to the construction of diagnostic features and the entire character matrices in paleontological studies. The two coding strategies should be distinguished following typical communication system engineering, because they serve dual purposes. With character matrices from six different vertebrate groups, we analyzed their information properties including source entropy, mutual information, and channel capacity. Estimation of channel capacity shows character saturation of all matrices in transmitting paleontological information, indicating that, due to the presence of noise, oversampling characters not only increases the burden in character scoring, but also may decrease quality of matrices. We further test the use of information entropy, which measures how informative a variable is, as a character weighting criterion in parsimony‐based systematic studies. The results show high consistency with existing knowledge with both good resolution and interpretability.

## Graphical abstract

Resembling a communication system, the fundamental problem of paleontological studies is reconstructing at present either exactly or approximately organisms living in another age. Information theory provides a new perspective in paleontological systematic studies.

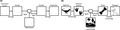

## 1 INTRODUCTION

Most extinct fossil organisms only preserved their morphology rather than macro‐biomolecules including DNA and proteins. Therefore, researchers need to convert the morphology of fossils into sequences, a series of scored morphological characters, for example, and analyze such sequences to identify each OTU (operational taxonomic unit, classification) and reconstruct their evolutionary history (systematics). However, unlike DNA or protein sequences coded by fixed alphabets (4 nucleotides and 20 amino acids), there is not a universal morphological alphabet that can digitize the morphology of extinct organisms into sequences. A practical and probably the most common way to convert morphology into sequences is constructing morphological characters matrices, which contain various OTUs and characters. According to the morphology of different OTUs, they are scored different states, usually 0 and 1, for different characters. The difficulties in constructing morphological characters have been realized early (Wilkinson, 1995), and many early attempts to propose methods/guidance in character construction are far from satisfactory (Estabrook et al., 1975; Hawkins et al., 1997; Sereno, 2007). The definition of “character” (in cladistics analysis) has also been discussed a lot (see review by Sereno, 2007) but is far from being universally applied.

Besides the most basic question of what a character is, discussions have been ongoing on whether to use giant matrices (Laing et al., 2018) or not (Simões et al., 2017), which anatomical structures should be represented by characters (Brocklehurst & Benevento, 2020), whether to combine morphological characters with molecular data and shape data (Nylander et al., 2004; Catalano et al., 2010), etc. Moreover, due to the incompleteness and distortions from preservation environments, most morphological character matrices can only be partially scored. If morphological characters are the most basic units in morphology‐based systematic studies, which resemble the nucleotides in DNA sequences and amino acids in proteins, analyzing character matrices under the framework of information theory may help to better understand those arguments.

The word information is repeatedly used in systematic studies (Cracraft, 1974; Farris, 1979; Mickevich & Platnick, 1989; Wilkinson et al., 2004; Sereno, 2007; Simões et al., 2017; Laing et al., 2018) but often it seems to be confused with data, signal, or its embedded semantic meaning. Few studies have connected information theory with systematic studies, especially for fossil‐based analyses. Similarly, during the early development of tele‐communication systems, even after the extensive applications of telegraph, telephone, and broadcast in 1940s, people did not formulize a complete theory of communication system engineering until information theory was proposed by Shannon (1948). Before constructing any communication system, it should be noticed that the signals themselves are irrelevant to their semantic meaning. Imagine a paleontologist and a local guide are working in a remote fossil locality, the guide stays in the camp and the paleontologist is looking for fossils. The paleontologist finds a dinosaur skeleton and needs tools from the camp to dig it out, but it takes too long to walk back. The paleontologist wants the local guide to bring with those tools. A smart way to do so may be to make an agreement with the local guide before leaving the camp as following: raising a red flag means the paleontologist needs fossil digging tools; raising a blue flag means he needs food. With such agreement, the paleontologist and local guide are communicating fairly efficiently, the only concern would be whether the local guide can see the color of the flag, but not the meaning of the color itself.

The mixture of signals and their semantic meaning can cause serious problems in communication, because exactly the same signal may have totally different meanings. This ignorance had brought difficulties in improving the quality of communication because no guidance existed to maximize the efficiency of coding information source or to minimize the influence of noises in communication channels.

## 2 PALEONTOLOGICAL SYSTEMATIC STUDY AS A COMMUNICATION SYSTEM

**FIGURE 1:**
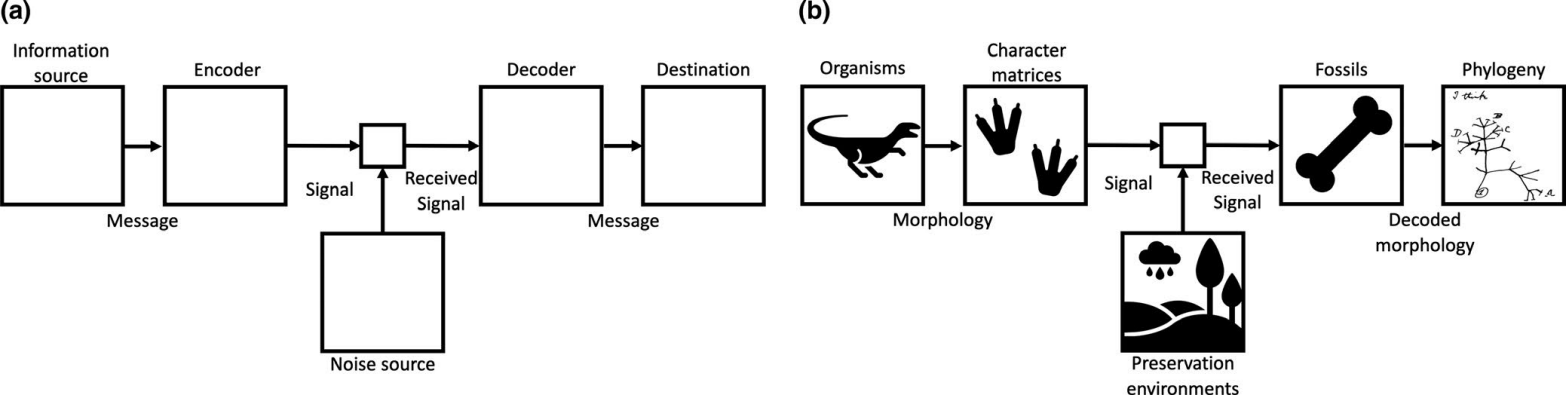
(a) Typical communication system modified from Shannon (1948); (b) Paleontological systematic studies in abstract

Shannon (1948) indicated that information is the decrease of uncertainty and a typical communication system can be divided into 5 parts, the information source, encoder, channel (which usually introduces noise), decoder, and the destination (Figure 1a). Shannon (1948, pp. 379) stated that “*The fundamental problem of communication is that of reproducing at one point either exactly or approximately a message selected at another point*.” Paleontological systematic studies share abundant similarities with a communication system (Figure 1b) and focus on reconstructing the evolutionary history of extinct organisms. Most modern communication systems such as telephone, email, and instant messaging apps are for communication in spatial domains, whereas paleontological systematic studies represent communication in the temporal domain. The original organisms can be treated as the information source, fossils discovered as the received message, and the preservation environments as noisy channels. Although some signals are either lost or distorted during preservation, we are interested in how much information is preserved and whether or how we can reconstruct those lost or distorted signals based on known ones. The encoder in Figure 1a encodes the original messages into signals, for example encoding “I need fossil digging tools” into a red flag as the example before, and the decoder does the *vice versa*. In paleontology, a widely used encoder is the morphological character matrix that encodes each OTU as a sequence of character states. The fundamental problem of paleontological studies is reconstructing at present either exactly or approximately organisms living in another age. Two questions must be answered to do so: (a) that how much information was in an organism or taxon? and (b) how much information can be preserved?

For the first question, vast publications have described the morphology of fossils in fine detail, but such description cannot be directly applied in paleontological systematic studies. As the morphological character matrix is probably the most common encoder in paleontology, the number of characters in it determines how many signals can be encoded, which draws the upper limit of how much information can be transmitted.

For the second question, although most fossils themselves are not well‐preserved, the preserved elements may help to reconstruct the missing parts. Many dinosaur species are named based on limited fossils, for example, *Deinocheirus* (Dinosauria, Theropoda) was firstly collected in 1965 (Kielan‐Jaworowska and Dovchin, 1966), when only forelimbs and several other fragments were discovered, then Ostrom (1972) recognized its affinity with ornithomimosaurs, which was later supported by Senter (2007), and finally the discovery of almost entire skeletons ended most arguments by Lee et al. (2014).

In communication system engineering, such processes are named source coding and channel coding; their differences are listed in Table 1. Source coding focuses on minimizing the cost at encoding all original messages. For example, Morse Code uses different lengths of codes to represent each letter in the alphabet, minimizing coding costs is attained by attributing the shortest code (a single dot) to the letter with the highest frequency in English (the letter *E*), whereas rarer letters such as *X*, *Y*, and *Z* have longer codes. On the other hand, channel coding is designed to resist noises in the preservation environments. The simplest but inefficient example of channel coding is repeated codes. If an information source is randomly sending 0 and 1 via a noisy channel that has a 30% chance to reverse the original message, thus any 0 or 1 received has a 70% chance to be correct. To resist such noise, the encoder repeats each message three times, which turns “0” into “000” and “1” into “111,” thus under maximum likelihood decoding principle that “000,” “100,” “010,” and “001” are decoded as “0” and others as “1.” The received message has a 78.4% chance to be correct (0.7^3^ + 3 × 0.7^2^ × 0.3 = 0.784), which is better than the original encoding method. However, repeated code is usually inefficient because in this example the encoding has tripled the cost but accuracy only improves 8.4%.

**TABLE 1:** Comparison between source coding and channel coding

	Source coding	Channel coding
Approximation	Source information entropy	Channel capacity
Redundancy	Discard	Introduce
Purpose	Increasing efficiency	Increasing robustness
Examples	Morse Code	Repeated codes

The joint source‐channel coding theorem (Shannon, 1948), also known as source‐channel separation theorem, shows that source coding and channel coding can be separated without influencing the other. If the channel capacity is strictly greater than source information entropy, noiseless communication can be achieved via sophisticated engineering, even in a noisy channel. In practice, the information encoder is often engineered into decoupled source and channel encoders to serve different purposes as in Table 1.

Similarly, the differences between source coding and channel coding have been realized and practiced in many paleontological systematic studies. In various studies including Nelson (1972) and Cracraft (1974), researchers have shown the differences between classification (Linnaeus classification and its variants) and systematics (phylogenetic classification, evolutionary classification, evolutionary systematics, etc.). Harrison (1993) emphasized the necessity of separating classification, corresponding to source coding, and systematics, corresponding to channel coding, in paleontological systematic studies. This separation is actually automatically applied in paleontological systematic studies, especially studies reporting new taxa, in which the characterization of the new taxon needs only few diagnostic features, whereas subsequent systematic analysis requires many.

**FIGURE 2:**
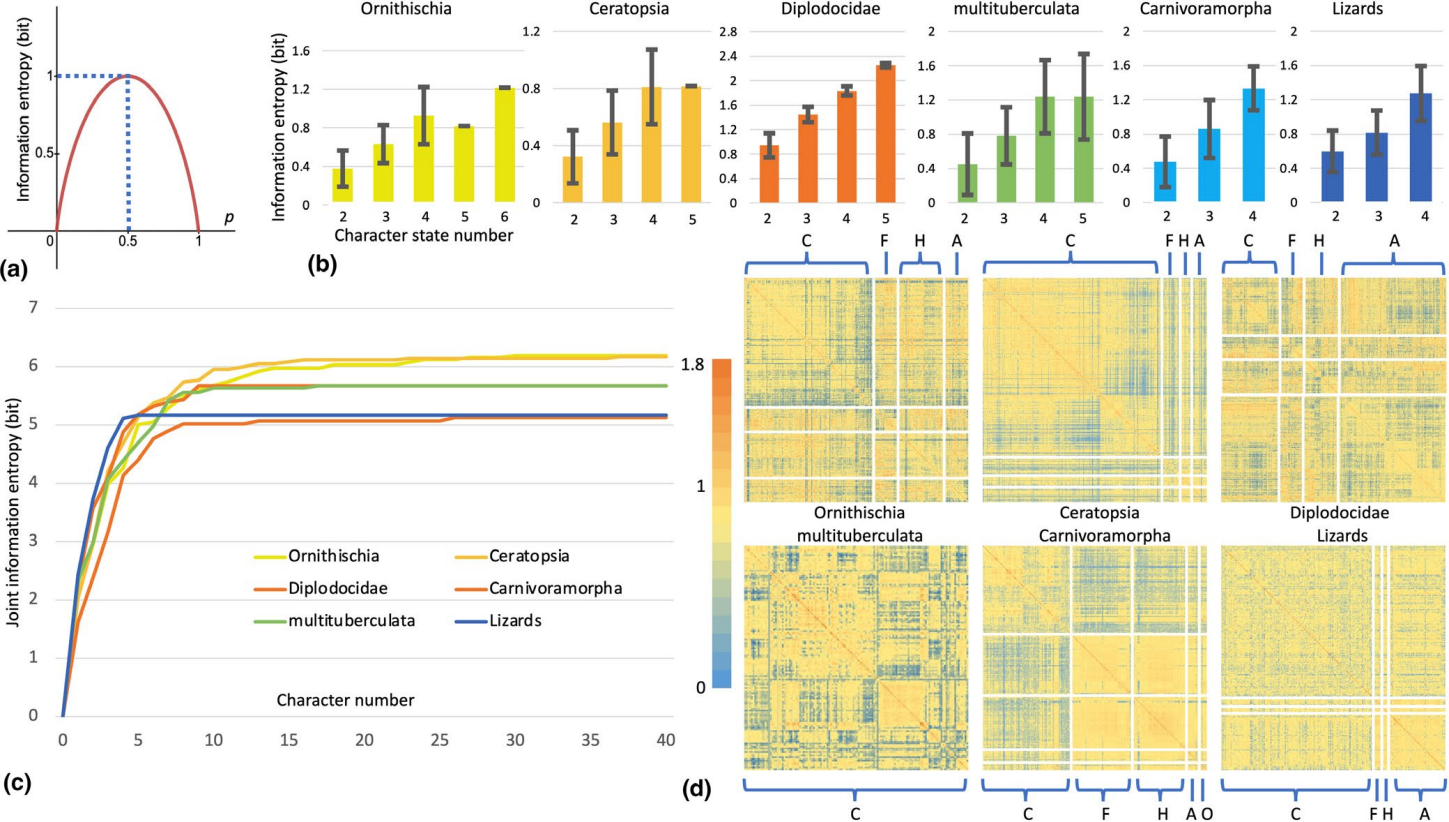
(a) Information entropy distribution of a binary variable; (b) information entropy of characters in different morphological character matrices, x‐axis: number of character states, y‐axis: average information entropy; (c) joint information entropy in different morphological character matrices, only first 40 characters are shown to save space; (d) mutual information distribution heat map in different morphological character matrices, orange: high mutual information, blue: low mutual information, anatomical parts abbreviations, C: crania, F: forelimb and pectoral girdle, H: hindlimb and pelvic girdle, A: axial elements, O: others

Morphological characters usually have two states which can be coded as 0 and 1. Although sometimes more states are available, multi‐state characters can always be split into several binary ones. A morphological character can be treated as a variable with discrete distribution on a group of organisms and we are interested in how much information is in a morphological character. Information is defined as the decrease of uncertainty (Shannon, 1948). If a character is scored the same among OTUs in a group, the information given by this character should be 0 because it does not decrease any uncertainty. The information given by a variable is its information entropy and can be calculated as follows:

(1) $$H=‐\sum p_{i}\log_{2}\left(p_{i}\right)$$

where H is the information entropy (measures in bit if the base of logarithm function is 2) and *p_i_* represents the probability of i‐th possible value of the source variable, putatively possible states of morphological characters in paleontological studies. With the change of distribution of character states, the information given by a character also changes and reaches its maximum at equal distribution. For a binary variable P with its values in probability of p and $\left(1‐p\right)$, its information entropy is as follows:

(2) $$H=‐p\log_{2}\left(p\right)‐\left(1‐p\right)\log_{2}\left(1‐p\right)$$

and this relationship between information entropy and probability is illustrated in Figure 2a. Consider the premaxillary teeth in Ceratopsia (Dinosauria, Ornithischia) as a morphological character, which is scored as 0 if not present and 1 if present. There are about 80 species of ceratopsians reported and 12 of them have premaxillary teeth, the information entropy of the premaxillary teeth character in Ceratopsia is 0.61 bit, which means an observation of this character in a ceratopsian species provides 0.61 bit information in average. However, it should be noticed that the distribution of character states is subject to change with new fossils, which will change its information entropy. With the increase of OTUs, the influence from newly added OTU decreases. If every binary character has an information entropy of 1 bit, namely, character states have equal distribution, *n* binary characters can classify $2^{n}$ taxa in the ideal situation. Table 2 is an example character matrix including 9 taxa and 3 scored binary characters (0 means absence and 1 means presence of a structure) to illustrate the differences between source coding and channel coding in paleontological systematic studies.

**TABLE 2:** Example character matrix showing source coding

Taxa	Character				
	Tail	Feather	Five digits	Scored sequence	Other characters
1	0	0	0	000	…
2	0	0	1	001	…
3	0	1	0	010	…
4	1	0	0	100	…
5	0	1	1	011	…
6	1	0	1	101	…
7	1	1	0	110	…
7.1	1	1	0	110	…
8	1	1	1	111	…

In the character matrix shown in Table 2, there are 9 taxa and only 3 scored binary characters. The sequences of taxa 7 and 7.1 are the same based on the given three characters, hence they cannot be distinguished without other characters being observed. If we would combine taxa 7 and 7.1 as a single OTU, all three characters have information entropy of 1 bit. Although these 3 binary characters are sufficient to distinguish 8 OTUs, they are far from enough to produce a resolved evolutionary cladogram. Usually in practice, the number of characters is much larger than the number of taxa in a character matrix and larger character matrices seems to be a trend in paleontological systematic studies (O’leary et al., 2013; Laing et al., 2018; Baron et al., 2017a). In Table 2, the construction of characters is not only insufficient to represent source information entropy, 7 and 7.1 are indistinguishable, but also vulnerable in systematic analysis. There are no redundant scored characters to resist noise or loss of data as the mutual information between each pair of characters is 0 bit if 7 and 7.1 are combined as a single OTU. For example, if the digit parts of the fossils are not preserved, taxa 1&2, taxa 3&5, taxa 4&6, and taxa 7&7.1&8 are indistinguishable because they are equally scored in the other two characters in the same states. Or for some reason, some fungus fossils associated with taxon 4 are erroneously identified as feathers, so it can be confused with taxa 7 and 7.1. From this simplified example, we can conclude that, to construct a comprehensive and robust character matrix, the sequences of character states should represent the source information entropy completely, and enough redundancy based on mutual information should be incorporated to minimize the influence of incomplete fossils and misidentification of character states.

Since there are many characters in a character matrix, we are interested in their mutuality that strongly influences the quality of character matrix. If two characters are strongly dependent, we can infer the state of a missing character according to observed state of the other, which may provide insight in dealing with incomplete specimens and dividing modules in mosaic evolution studies. In previous content, we show that the information of a variable is defined as the uncertainty it decreases, and thus, the uncertainty of a variable *A* given by another variable *B* is the mutuality between them, the mutual information.

(3) $$I\left(A,B\right)=H\left(A\right)‐H_{B}\left(A\right)=H\left(B\right)‐H_{A}\left(B\right)=H\left(A\right)+H\left(B\right)‐H\left(A,B\right)$$

where $I\left(A,B\right)$ is the mutual information, $H\left(A\right)$, $H\left(B\right)$, and $H\left(A,B\right)$ are the information entropy of variable *A*, *B*, and their joint distribution, respectively, $H_{B}\left(A\right)$ and $H_{A}\left(B\right)$ are the conditional entropy of these two variables. If the marginal and joint distributions of variable *A* and *B* are known:

(4) $$I\left(A,B\right)=\sum_{a}\sum_{b}P\left(a,b\right)\log_{2}\frac{P\left(a,b\right)}{P\left(a\right)P\left(b\right)}$$

In Table 2, we can calculate the mutual information between each pair of characters and the results are 0 for all pairs (taxa 7 and 7.1 are treated as a single OTU). The zero mutual information in this designed character matrix indicates that the tail, feather, and five digits characters are independent from one another, namely, the knowledge of a character in a taxon does not decrease the uncertainty from another character. The lack of dependence also explains the vulnerability of diagnosis when a character cannot be observed.

Mutual information and joint information entropy can be further generalized to multiple variables. We can calculate the joint information entropy of the entire character matrix according to the joint distribution of character states as following:

(5) $$H\left(A_{1},A_{2},\ldots \ldots ,A_{n}\right)=‐\sum_{a_{1}}\ldots \ldots \sum_{a_{n}}P\left(a_{1},a_{2},\ldots \ldots ,a_{n}\right)\log_{2}P\left(a_{1},a_{2},\ldots \ldots ,a_{n}\right)$$

where $A_{i}$ represents one of the multiple variables. To simplify expression (5), we use $s_{\mathrm{ij}}$ to represent the *j*‐th unique sequence of first *i* characters in the matrix. For example, in the character matrix given by Table 2, $s_{21}=00$ and $s_{33}=010$.

(6) $$H\left(\text{first i characters in matrix}\right)=‐\sum_{j}P\left(s_{i}\right)\log_{2}P\left(s_{i}\right)$$

A study by Baron et al. (2017a) proposed a significantly different dinosaur phylogeny, in which Theropoda and Ornithischia are sister groups, forming the Ornithoscelida, and Sauropodomorpha and Herrerasauridae form Saurischia as sister group to Ornithoscelida. In a comment to Baron et al. (2017a), Langer et al. (2017) recovered the “traditional” topology of dinosaur phylogeny with a dichotomy of Ornithischia and Saurischia, the latter including Theropoda, Sauropodomorpha, and “Herrerasauridae.” The subsequent reply by Baron et al. (2017b) mentioned that, “*Langer et al*.*, identify numerous disagreements in terms of character scoring and suggest changing approximately 2,500 scorings, around 10% of the character data*.” Given that there are only tiny differences between methods (Langer et al., 2017, supplementary information), it is clear that the incongruence of original character scoring had led to the contrasting results, but not the algorithm used to reconstruct the phylogeny. Both sides of authors tried to score the vast number of morphological characters in the matrix (“*457 anatomical features scored for 74 early dinosaurs and close relatives*”) as accurately as possible, but rescoring a single character of a single taxon, *Pisanosaurus mertii*, has led to a considerably different result (Baron et al., 2017b, Figure 1). This vulnerability reflects the fact that this morphological character matrix cannot provide robust results, although the taxon and character numbers in these studies are larger than many previous studies. Comparably, even before Shannon proposed the information theory, communication engineers have designed codes, for example, Morse Code, and found factors influencing transmission quality in noisy channels (Nyquist 1924, 1928). A general problem had been realized that blindly increasing the power of signal cannot improve communication quality after certain thresholds in noisy channels.

In typical digital communication systems, all messages are coded in 0 and 1 for transmission. The frequency of the transmitter is defined as how many changes can be made during 1 s with unit Hz. With the increase of frequency, more signals can be sent within a given time span thus more information can be transmitted in ideal situation. According to the similarity between communication system and paleontological systematic studies discussed before, the concept of frequency in communication systems in the spatial domain can be transcribed in paleontological systematic studies in the temporal domain as the number of characters, namely, bandwidth. Intuitively, if every fossil specimen is complete and undeformed (noiseless channel), increasing the number of morphological characters can describe their morphology in finer detail, which correlates the trend of using giant matrices currently. However, such positive correlation only partially holds under noisy circumstances, as noises also increase with the increase of bandwidth. Channel capacity, the maximum rate of reliable communication, is limited by the presence of noises even with arbitrarily large bandwidth. Shannon (1948) shows the relation between channel capacity and bandwidth of Additive White Gaussian Noise (AWGN) channel:

(7) $$C=B\log_{2}\left(1+\frac{S}{N}\right)$$

where C is the channel capacity (bit), B is the bandwidth (range of available frequency in wireless communication and number of characters in this study), S and N are the power of signal (scored characters) and noise (unscored characters), respectively. AWGN channel assumes that noises are (a) additive to the system but not intrinsic; (b) white, uniform power across frequency; (c) Gaussian, in normal distribution in the time domain. AWGN is a basic model simplifying many natural random processes as a whole. Because the unscored character states in a matrix are controlled by many complicated factors and the extreme difficulty of modeling most non‐Gaussian systems, AWGN channel offers convincing results at this stage. It should be noticed that $N=n_{0}B$, where $n_{0}$ is the noise density per bandwidth unit, and thus, the maximum channel capacity is approximate $1.44B\frac{S}{N}=1.44\frac{S}{n_{0}}$ bits in AWGN channel.

## 3 MATERIAL AND METHODS

In this study, we calculated the information properties and run parsimony‐based phylogenetic analyses on character matrices from 6 different vertebrate groups: Ornithischia (Han et al., 2017), Ceratopsia (Yu et al., 2020), Diplodocidae (Tschopp & Mateus, 2017), multituberculata (Wang et al., 2019), Carnivoramorpha (Spaulding & Flynn, 2012), and lizards (Tschopp et al., 2018). We first quantified the information entropy of each character in six matrices. To investigate the mutuality among characters, the mutual information in each character matrix is calculated. To access the differences between source coding and channel coding, we then calculated the joint information entropy of first $n\left(n\leq \text{total character number}\right)$ characters. Last, we use the model of AWGN discrete channel to estimate the channel capacity of fossil preservation environments.

For characters with missing data in the character matrices, we estimate the missing parts to have equal distribution among different states. For example, a binary character is scored 0 in 20% OTUs, 1 in 40% OTUs, and missing in 40% OTUs, the estimated distribution would be 0 in 20% + 0.5 × 40% = 40% and 1 in 40% + 0.5 × 40% = 60%. We also calculate those values without the estimation of missing data as a reference. Calculation is done by custom Python 3.7 scripts.

Phylogenetic analysis was done in TNT 1.5 using traditional search with default settings (Goloboff & Catalano, 2016), in which implied weighting used *k* = 3 and12. The strict consensus tree was appended to the last of tree list in each group. CI and RI are discussed for the strict consensus trees.

## 4 RESULTS

The distribution of character information entropy (Figure 2b) shows that characters with more states tend to have higher information entropy, indicating those multi‐state characters generally introduce more information in systematic studies. Among characters with the same number of states, the information entropy still varies a lot in most datasets.

Six matrices show consistent pattern in their joint information entropy (Figure 2c, only first 40 characters are shown). For the first few characters, the joint information entropy increases fast to approximate the source information entropy, which is the upper limit of joint information entropy, and the majority of characters serve in channel coding as they do not contribute to the source coding much. The curves of joint information entropy show that only a few characters are required to distinguish each OTU (classification) and the majority of characters in the matrices are for channel coding (systematics).

The mutual information within the 6 matrices is calculated (Figure 2d) to test the mutuality between characters. Due to the existence of missing data, the diagonal line numbers showing mutuality between any character and itself do not strictly correspond to the character's information entropy but are still generally higher than other areas of the heat maps. The distribution of mutuality seems to have no pattern in most matrices. After reordering and partitioning characters by anatomical structures (crania, pectoral girdle and forelimb, pelvic girdle and hindlimb, axial bones, and others), some parts exhibit relatively high mutuality, for example, the forelimbs and hindlimbs of Carnivoramorpha (Spaulding & Flynn, 2012) show both higher inter‐ and intramutuality than other anatomical structures.

**FIGURE 3:**
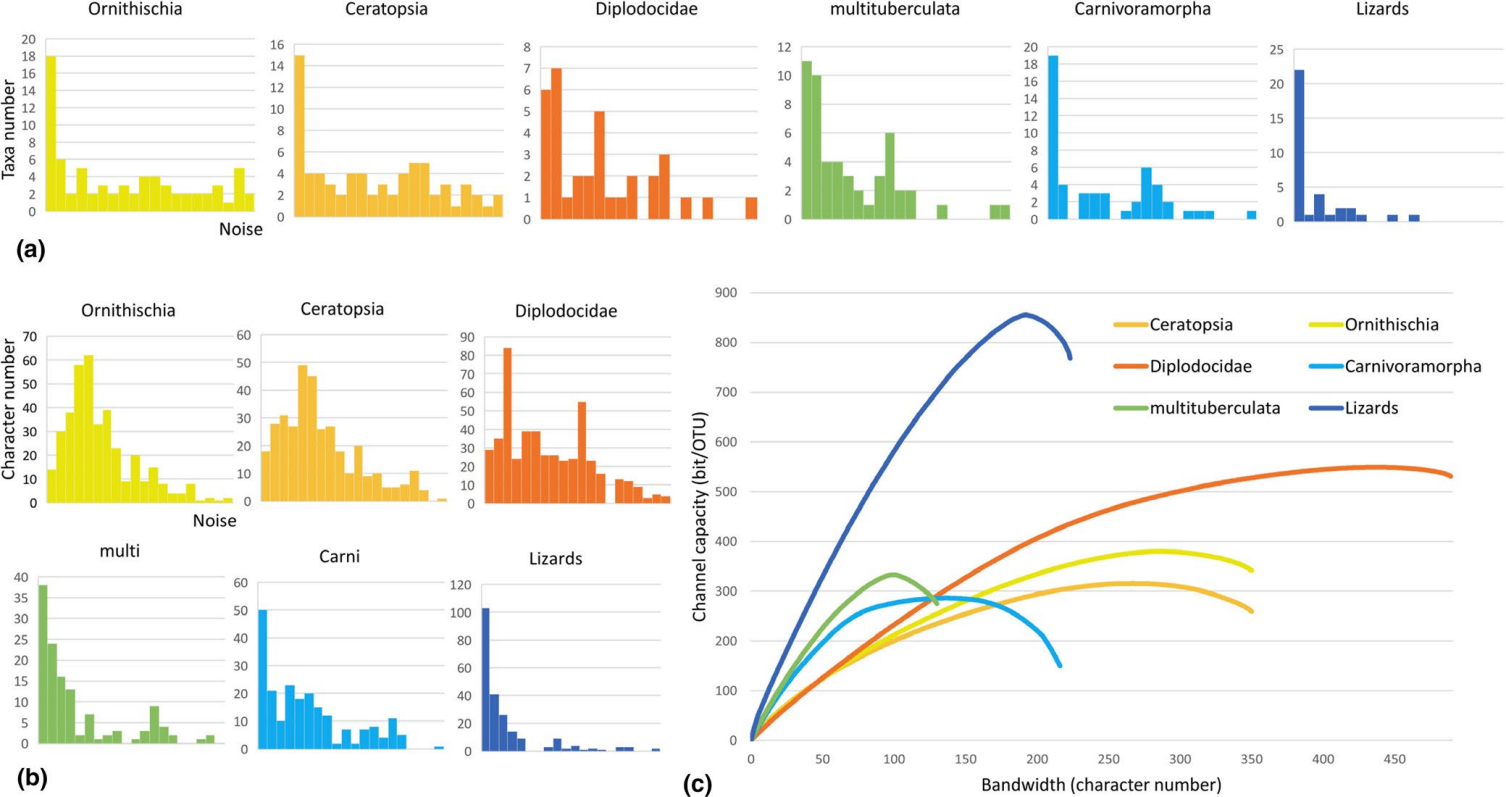
(a) noise power distribution in taxa domain; (b) noise power distribution in character domain; (c) channel capacity and bandwidth in character matrices

The distributions of noise power in the taxa domain and the character domain are shown in Figure 3a and b, respectively. The results show saturation in channel capacity when increasing bandwidth, the number of characters (Figure 3c). Different character matrices reach the maximum channel capacity when having 62.5% (multituberculata) to 89.7% (Diplodocidae) of total characters.

## 5 DISCUSSION

### 5.1 Information source

No matter what algorithm is being used in systematics studies, the common aspect is using sequences (DNA, amino acid, and morphological characters) to characterize organisms and to interpret their evolutionary history. With fixed alphabets, DNA and protein sequences resemble digital signals in modern communication systems, while morphology of fossils is more like analog signals. Therefore, the process of character construction is the same as sampling digital signals from analog signals, and meanwhile, the probably infinite original information entropy of fossil morphology is converted into finite entropy, represented by hundreds to thousands of morphological characters, that can be more easily compared. More morphological characters usually describe organisms more completely, but it is extremely difficult to measure how completely the character matrix characterizes the overall morphology of a group of organisms. There is not a standard guidance on character selection, and many characters in matrices are selected because researchers believe they carry morphological information. The interrelationship among morphological characters and how they connect to the overall morphology remains uncertain. At least from the results of mutual information and channel capacity against bandwidth (the number of characters), we show that the dependence between characters and different anatomical structures is complex, and current morphological character matrices seem to reach the saturation of characters already. Shannon (1949) proposed the Sampling Theorem (also known as Nyquist‐Shannon Sampling Theorem because early work was done by Nyquist 1924, 1928), which bridges the continuous signals and discrete signals. With a continuous signal source of a finite bandwidth, Sampling Theorem shows the lowest sample rate to capture all information, which is twice the rate of highest rate of original signals. As the connection between bandwidth in typical communication systems and character number of paleontological systematic studies is discussed before, Sampling Theorem may be a bridge between raw morphology and morphological characters.

However, the saturation of channel capacity (Figure 3c) does not necessarily mean those morphological character matrices fully represent the entire morphology of fossil specimens. Such saturation only shows these matrices cannot sufficiently transmit the sampled morphological information in themselves while some other information may be left as the sampling of characters are strongly biased. The morphological matrix of multituberculata (Wang et al., 2019) comprises only characters from the cranial region, but the postcrania of those organisms also have information.

With the wide applications of advanced imaging techniques such as CT (computed tomography) scan, it is feasible to capture the complete morphology of fossil specimens without destruction. The unprecedented amount of data may be the stepstone to establish the connection between analog morphological data and digital character data. A standard workflow may be possible to morphological studies under the facilitation from information theory and high‐resolution imaging.

### 5.2 The properties of the channel (bandwidth, channel capacity, noise)

In this study, we use one of the most basic models, AWGN channel, to mimic preservation environments with limited explanation. AWGN channel requires that noises have uniform power in frequency domain and Gaussian distribution in time domain. Treating the character number as bandwidth, then the characters probably correspond to the frequency domain in a typical communication system and the OTUs to the temporal domain. This model sounds natural based on the model in Figure 1 as every organism ever lived on earth was a message sent, and fossils are a small fraction received. However, in the character matrices analyzed here, many OTUs are scored based on multiple specimens, therefore result in the aggregation of scored characters in the first few columns in Figure 3a. For the temporal domain/OTUs, the noises derived from natural preservation and are controlled by many factors, so it is probably fair to use AWGN channel model for both simplification and convenience.

From the estimation based on AWGN channel model (Figure 3c), all character matrices show saturation of characters. The basic explanation of saturation is that with the increase of bandwidth, the noise also increases. Incompleteness, deformation, and misidentification are common among the fossil specimens. If the nature of the paleontological information channel is noisy, we cannot expect to efficiently transmit paleontological information without channel coding. Moreover, the time costs in both encoding and decoding have to be considered when facing extremely giant character matrices.

### 5.3 Character matrix construction and weighting

The construction of (morphological) character matrices is central to systematic studies and has been discussed extensively. In this study, we make the initial attempt to quantify the information in existing morphological character matrices for the first time. Many results show consistence to common understanding of morphological characters, including different characters having different amount of information, mutuality existing among characters, more characters usually carrying more information, etc. Besides, we also propose that the information entropy of each character can be used as their weights in phylogenetic analysis.

As the information entropy represents how informative a character is, it may be a candidate of character weighting in phylogenetic analysis. Most researchers agree that some kinds of weighting should be applied in systematic analysis and equal weighting is one of the weighting methods (Farris, 1969; Sereno, 2007). Based on the successive weighting proposed by Farris (1969), Goloboff (1993) proposed implied weighting and extended implied weighting (Goloboff, 2014). These weighting methods refine the weights of different characters to reduce homoplasy. However, Congreve and Lamsdell (2016) indicated that implied weighting is not consistent with the idea of parsimony and increase both correctly and incorrectly resolved nodes with simulated datasets. The wide use suggests that implied weighting and its variants probably provide a direction in reconstructing better resolved trees, but neither the theoretical basis nor its utilization answers the core question of how much information is in each character and may fail when working with character matrices with too many homoplastic characters.

Birds and modern mammals are both endothermic, covered with filaments rather than scales, having four‐chamber hearts, etc. If we would deliberately sample too many characters describing these features, the conclusion could easily be forced into that birds are mammals, and many synapomorphies between birds and other reptiles, for example, the presence of sclerotic rings, can be recovered as homoplasy. Fortunately, there are many other lines of evidence, which mean more information, showing that birds are more closely related to modern reptiles than modern mammals. The morphology and physiology of birds, the genetic data, and the fossil records all indicate that these similar features between birds and mammal are results of convergent evolution. It is not reasonable to refute that birds are dinosaurs with considerable fewer features against the overwhelming evidence from fossils, molecular biology, anatomy, and many other aspects. However, such biased sampling of characters can be hard to recognize in extinct groups with only limited fossil materials and implied weighting may even strengthen such bias. But information theory may discover those biased sampling. If such a character matrix exists, since its biased sampling, the mutual information among characters would be high and the channel capacity may not be saturated by the number of characters, because there is only little information represented by biased sampled characters.

Successive weighting, implied weighting and their variants require an initial weight or an existing tree topology, whereas information entropy weighting only depends on the information entropy in each character. In matrix construction, the choice of characters is often extremely biased toward cranial characters in vertebrate paleontology studies (Figure 2d). In the six datasets we analyzed here, the proportion of cranial characters range from 40.7% to 100% with an average of 63.2%, which immediately shows that some parts are considered to have more morphological information (or to be “more important”) than others in systematic studies.

Kälersjö et al. (1999) studied plant nucleotides data and their results showed that fast evolving and highly homoplastic third codon positions, contrary to traditional thought, have the strongest phylogenetic information, and they also suggest that the frequency of change should be used as in character weighting and selection. Although these authors tried to quantify the information in different nucleotide sites, that is, molecular characters, they did not provide an explanation on how they define information/informative sites.

**FIGURE 4:**
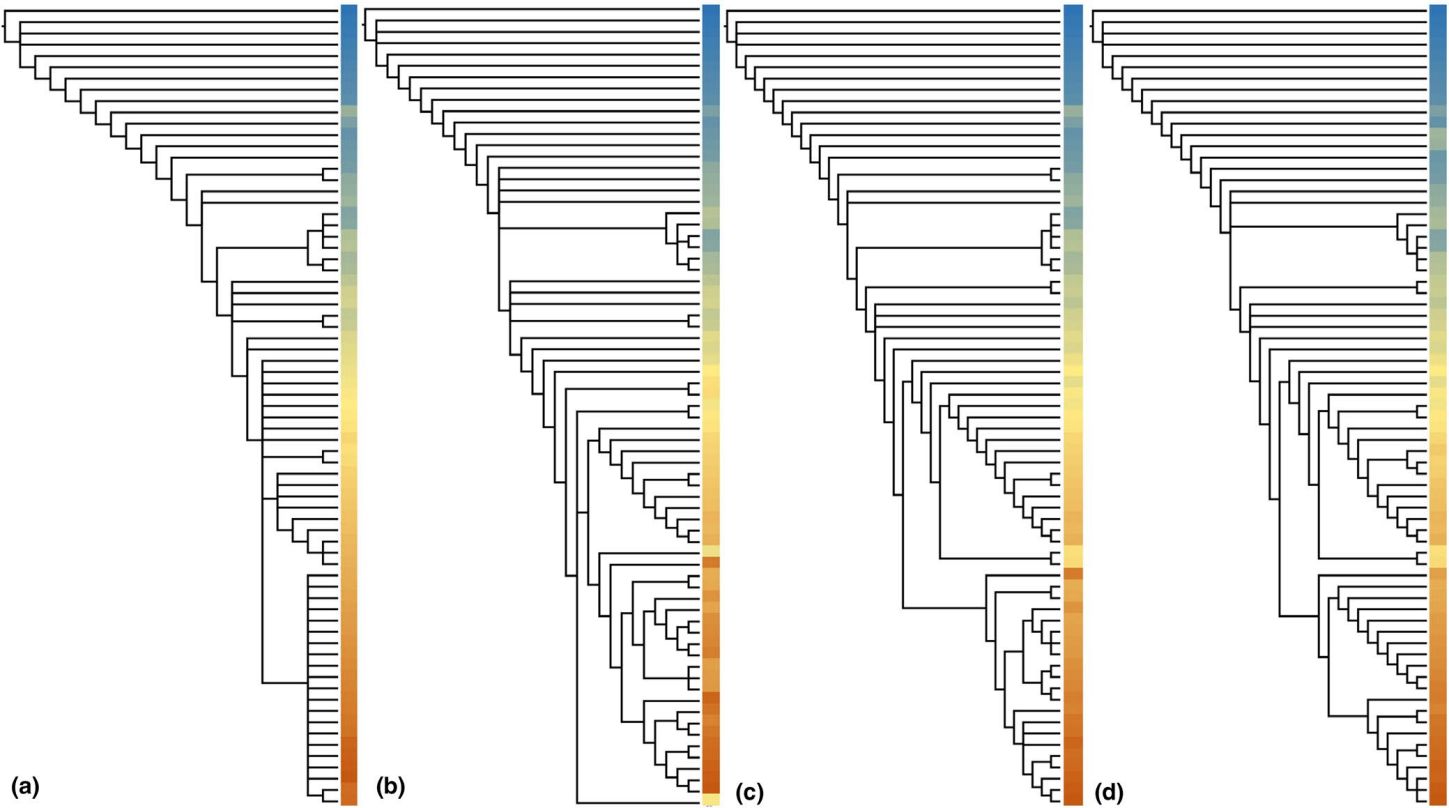
Tree results of Ceratopsia. (a) Equal weighting; (b) implied weighting (*k* = 3); (c) implied weighting (*k* = 12); (d) information entropy weighting. Colored columns on the right side of trees represent OTUs and their color gradients correspond to the taxa order in the original character matrix

We tested the results from equal weighting, implied weighting (*k* = 3&12), and information entropy weighting of six matrices analyzed before. Ceratopsia are illustrated in Figure 4. To save space and show the differences among trees, colored columns replace the OTU names on the right side of trees and color gradients correspond to the taxa order in character matrix. Detailed phylogenetic results are provided online at https://doi.org/10.5061/dryad.8sf7m0cnc. Generally, they show unexpected consistence between both equal weighting and implied weighting, but slight differences are common. The CI (consistence index) and RI (retention index) are also calculated for the most parsimonious tree of each group in Table 3. The CI of entropy weighting is generally slightly lower than other methods, and RI is slightly higher, suggesting that more homologous characters are suggested and the trees fit better for entropy weighted characters.

**TABLE 3:** CI and RI of different morphological character matrices

Index	Weighting	Ornithischia	Ceratopsia	Diplodocidae	multituberculata	Carnivoramorpha	Lizards
CI	Equal	0.369	0.518	0.345	0.327	0.261	0.246
	Implied (*k* = 3)	0.352	0.502	0.344	0.44	0.267	0.241
	Implied (*k* = 12)	0.352	0.511	0.345	0.446	0.270	0.246
	Entropy	0.343	0.498	0.331	0.433	0.262	0.236
RI	Equal	0.71	0.844	0.526	0.795	0.578	0.468
	Implied (*k* = 3)	0.688	0.833	0.525	0.748	0.590	0.452
	Implied (*k* = 12)	0.688	0.839	0.525	0.754	0.596	0.467
	Entropy	0.703	0.846	0.530	0.761	0.61	0.478

## 6 CONCLUSION

Under the framework of information theory and communication system engineering, we show that the information entropy, which measure how informative a character is, varies a lot in different characters. Characters with more states have significant higher information entropy than binary characters. Mutuality between characters does not show clear patterns except for limited body parts. All six character matrices analyzed in this study have oversampled characters that lead to saturation in channel capacity. Last, information entropy, without any prerequisites, can be used as a criterion for character weighting in systematic studies.

## CONFLICT OF INTEREST

All authors declare that they have no conflicts of interest.

## AUTHOR CONTRIBUTIONS

**Congyu Yu:** Conceptualization (lead); Data curation (lead); Formal analysis (lead); Investigation (lead); Methodology (lead); Project administration (lead); Resources (lead); Software (lead); Supervision (lead); Validation (lead); Visualization (lead); Writing‐original draft (lead); Writing‐review & editing (lead). **Qigao Jiangzuo:** Conceptualization (equal); Data curation (equal); Formal analysis (equal); Investigation (equal); Methodology (equal); Project administration (equal); Resources (equal); Software (equal); Supervision (equal); Validation (equal); Visualization (equal); Writing‐original draft (equal); Writing‐review & editing (equal). **Emanuel Tschopp:** Conceptualization (equal); Data curation (equal); Formal analysis (equal); Investigation (equal); Methodology (equal); Project administration (equal); Resources (equal); Software (equal); Supervision (equal); Validation (equal); Visualization (equal); Writing‐original draft (equal); Writing‐review & editing (equal). **Haibing Wang:** Conceptualization (equal); Data curation (equal); Formal analysis (equal); Investigation (equal); Methodology (equal); Project administration (equal); Resources (equal); Software (equal); Supervision (equal); Validation (equal); Visualization (equal); Writing‐original draft (equal); Writing‐review & editing (equal). **Mark Norell:** Conceptualization (equal); Project administration (equal); Supervision (equal); Writing‐original draft (equal).

## DATA AVAILABILITY STATEMENT

Phylogenetic results of Ornithischia, Ceratopsia, Diplodocidae, multituberculate, Carnivoramorpha, and lizards, by equal weighting, implied weighting (*k* = 3 and 12), and information entropy weighting are presented in.nex files. The strict consensus tree is appended in the end of trees in each file. The phylogenetic files can be found online at https://doi.org/10.5061/dryad.8sf7m0cnc.
